# Confidence Score: The Forgotten Dimension of Object Detection Performance Evaluation

**DOI:** 10.3390/s21134350

**Published:** 2021-06-25

**Authors:** Simon Wenkel, Khaled Alhazmi, Tanel Liiv, Saud Alrshoud, Martin Simon

**Affiliations:** 1 Marduk Technologies OÜ, 12618 Tallinn, Estonia; simon.wenkel@marduk.ee (S.W.); tanel.liiv@marduk.ee (T.L.); martin.simon@marduk.ee (M.S.); 2 National Center for Robotics and Internet of Things Technology, Communication and Information Technologies Research Institute, King Abdulaziz City for Science and Technology—KACST, Riyadh 11442, Saudi Arabia; srshoud@kacst.edu.sa

**Keywords:** computer vision, deep neural networks, object detection, confidence score

## Abstract

When deploying a model for object detection, a confidence score threshold is chosen to filter out false positives and ensure that a predicted bounding box has a certain minimum score. To achieve state-of-the-art performance on benchmark datasets, most neural networks use a rather low threshold as a high number of false positives is not penalized by standard evaluation metrics. However, in scenarios of Artificial Intelligence (AI) applications that require high confidence scores (e.g., due to legal requirements or consequences of incorrect detections are severe) or a certain level of model robustness is required, it is unclear which base model to use since they were mainly optimized for benchmark scores. In this paper, we propose a method to find the optimum performance point of a model as a basis for fairer comparison and deeper insights into the trade-offs caused by selecting a confidence score threshold.

## 1. Introduction

Object detection is still a hot topic in academia and industry. The term “object detection” yields ca. 33,400 search results on Google Scholar (excluding patents and citations) and arXiv.org lists 410 papers uploaded (title and abstract searched) for 2020. Considering that many new deployment scenarios of robots become feasible beyond tech/lab demos, the importance of reliable and robust object detection will increase further. Applications in e.g., medical sciences or maintenance identification of e.g., aircraft parts impose similarly high requirements for reliable and robust object detection.

Furthermore, in remote sensing applications reliability and robustness are increasingly critical as deploying object detection on-board satellites is becoming a necessity. Having an updated mosaic of the Earth’s surface with a frequency of once per hour and ground sample distance (GSD) of 5m would require over 1000 petabytes of data per day [1]. Transmitting this to ground-stations over telemetry is simply infeasible [2]. Thus, it is required to have only metadata updates of object detection, pattern recognition and anomalies instead of full-scale raw data transmission. Sensor development has led the way to ground structures detection [3] and analysis from remote sensing satellite imagery data. One example of this is bridge dislocation analysis, where a high degree of displacement detection precision is achieved [4,5]. This gives promise for remote sensing maintenance analysis for power grid and oil/gas pipelines as well. However, these applications have minimum requirements with respect to model robustness while maintaining deployability on edge devices. Instance segmentation is generally preferred in high-aspect-ratio object detection, which is often prevalent in these applications; however, in some circumstances, where computational power requirements are limited (e.g., on-board processing) or there is a lack of datasets classical object detection must do be sufficient enough.

On the research side, there is a clear focus on new model architectures, loss metrics (e.g., [6,7]) and loss functions (e.g., [8,9,10,11]). These are proposed and published constantly. However, benchmark datasets used in publications are not growing despite a growing number of datasets covering the task of 2D (e.g., [12,13,14,15,16]) and 3D object detection (e.g., [17,18,19,20,21,22,23]). Object detection models are primarily evaluated on very few datasets [24]; foremost they are evaluated on Pascal VOC [25] and COCO (Common Objects in Context) [26]. The former seems to be used for some form of legacy compliance whereas the latter seems to be used as the main evaluation baseline. Other datasets for e.g., remote sensing or autonomous driving are often not used in any ablation study—the KITTI dataset [27] might be an exception here but is mainly used for semantic segmentation.

At some point the research is evaluated for usage in industry. In practice, selecting a new model for further, application-specific evaluation is usually based on one or a combination of multiple of these considerations:

- (benchmark) performance metrics (e.g., COCO evaluation results),
- object size or class-specific performance metrics,
- inference speed (e.g., FPS as a function of batch size),
- computational cost/power consumption (e.g., for edge devices),
- source code license.

This often boils down to the usage of provided example scripts and pre-trained weights to either evaluate a model for scenarios with similar data to what it was trained on or retraining it for different datasets/tasks. When visualizing a model’s output, it becomes apparent that a model might actually either produce many false positives or misses a large number of objects (false negatives). Karol Majek provides a plethora of model performance examples on a 30 min recording of a drive through Warsaw, Poland (https://www.youtube.com/c/KarolMajek/videos, accessed on 6 February 2021).

Among other causes, the number of false positives/negatives is highly dependent on the confidence score threshold chosen. Frequently, the reference confidence score threshold used is not provided in the paper accompanying a model but must be inferred from the source code. Unfortunately, these confidence score thresholds are ridiculously low (<5%; <25% not uncommon) when comparing them to best practices in other fields of data science and machine learning. We may argue that the confidence score does not represent a model’s certainty in a strict probabilistic sense but many people, especially third-party users without expert knowledge, understand it this way.

In the fields of robotics or in medical applications, the consequences of an unreliable model for object detection are likely to be significantly more severe than e.g., in a deployment scenario that involves object detection for an online retail store [28,29,30]. Similarly, when considering on-board processing on satellites or other edge devices, consequences of large amounts of false positives and false negatives may render a product or an application pointless. Therefore, the number of false positives should be as low as possible—ideally approaching zero, and the number of false negatives should be zero as an object missed in e.g., several frames in a row might cause severe damage. Given an open-world deployment scenario, the trade-off between false positives and false negatives is not only about mitigation using constant retraining of a models deployed, but about finding a suitable confidence score threshold to mitigate adverse behavior in deployment scenarios.

**Figure 1:**
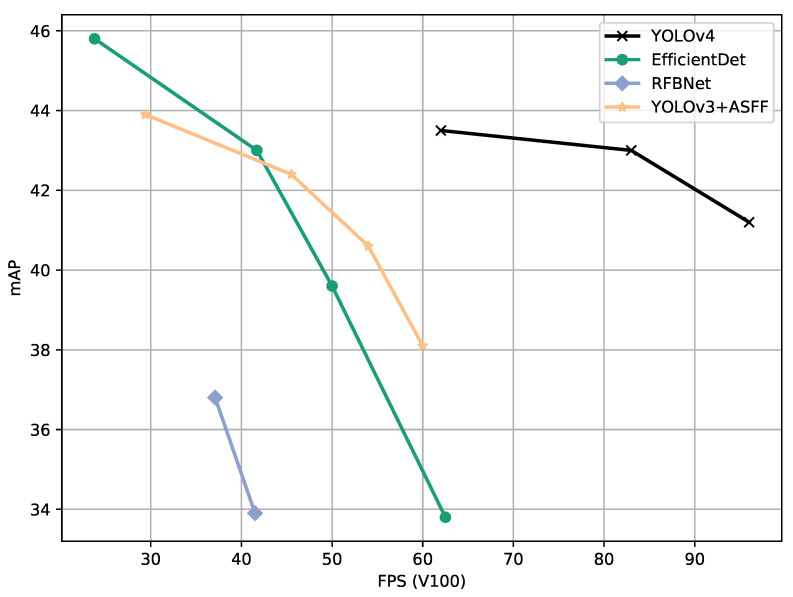
Example of a typical graphical model comparison on the COCO test set (data from Bochkovskiy et al. (2020) [31]).

Therefore, we evaluate a large subset of state-of-the-art models performance at different confidence score thresholds, using the standard COCO metrics as well as Probability-based Detection Quality (PDQ) [30], and propose a method for finding the optimal confidence score for a model as a basis for a fairer model comparison than what is commonly used (Figure 1). In other words, we propose a method to find the optimal operating point of a model that could be used to decide what model to choose and what to expect if the threshold is lowered or increased.

## 2. Experimental Setup

### 2.1. Dataset Selection

The selection of evaluation metrics (Section 2.3) requires that a dataset provides data suitable for instance segmentation and not just (horizontal) bounding boxes as this is required by the PDQ.

The COCO dataset is one of the most used and evaluated datasets for object classification DNN architecture and hyperparameter tuning benchmarks. Previous research indicates that the COCO dataset is well-suited for model robustness and efficiency analysis [32]. Since the COCO dataset could be considered to be the reference dataset for object detection and provides annotations for instance segmentation as well, we consider it necessary to use it as a basis. Moreover, almost all model architectures are provided with pre-trained weights on this dataset. This allows for a large selection of models to investigate the impact of the selection of the confidence score thresholds.

Although there is an abundance of image datasets of objects in forward looking setups (e.g., the COCO dataset), there is scarcity of over-head datasets usable for specific real-life and real-time applications on satellites, aircraft and UAV-s. The subset of datasets which provide annotations for instance segmentation as required by the PDQ metric. Therefore, the second dataset selected is the TTPLA (Transmission Towers and Power Lines Areal images) [13] dataset. This dataset contains aerial images captured from an UAV showing transmission towers and power lines. Therefore, it can be seen as a proxy for multi-purpose object detection on aerial images. With respect to UAV navigation and control it can be seen as a dataset for robust detection of obstacles to prevent damage caused by an UAV. Similarly, it can be seen as an example for inspection work carried out by UAVs. Furthermore, power lines have a high aspect ratio making this particularly challenging for object detection.

### 2.2. Model Selection

#### 2.2.1. COCO

Models are selected differently for both datasets. Models used for evaluation on the COCO dataset are selected to evaluate a good assortment of state-of-the-art models whereas TTPLA comes with a few reference models only.

Most object detection models are available pre-trained on the COCO dataset. Therefore, we choose the COCO val2017 subset as our evaluation basis. These pre-trained models are likely to be trained on the validation set. However, reported performance differences between val2017 and test2017 seem to be rather small, and therefore we can use this as a proxy. We selected a subset of state-of-the-art object detection models based on the following criteria:

- state-of-the-art results promised in accompanying publications
- deployed commonly in industry and academia
- small models suitable for deployment on edge device
- large models with highest scores despite high hardware requirements

These selection criteria lead to a list of selected models covering a broad range of different model architectures, approaches and deployment purposes (Table 1). Some models may use NMS (Non-Maximum Suppression) or SoftNMS [33] to output only the most suitable boxes whereas others do not. Some models, e.g., ones using SSD (Single Shot Detector) [34] are limiting their detections per image to 100 bounding boxes by default and therefore are matching the max. number of bounding boxes considered by the COCO API for evaluation.

#### 2.2.2. Models for Selected for Evaluation on TTPLA

We use the reference models provided by the TTPLA publication for resolutions of 550 × 550 and 700 × 700 pixels as our evaluation baseline. These reference models use YOLACT [45] with ResNet backbones [46]. Moreover, NanoDet and five models with different backbones for Faster R-CNN heads are trained for 150 epochs on TTPLA (resolution 700 × 700 px) for a better comparison. We used default settings provided by NanoDet and Faster R-CNN models available via detectron2. No mitigation of class imbalances and additional image augmentation during training was implemented.

### 2.3. Evaluation Metrics

Two metrics are used to evaluate each model’s performance on the COCO dataset. This includes the standard COCO evaluation metric as well as the PDQ. On the TTPLA dataset only the more relevant PDQ metric is used.

With respect to the COCO evaluation metric, we would like to point out a few important things. The most important thing is that the evaluation function sorts detections per class by confidence score and considers only the 100 best (highest confidence score) bounding boxes per image for evaluation. Therefore, it is clear already that if a model outputs a rather large number of bounding boxes with low confidence scores, then this is not penalized by the evaluation function.

Due to the nature of the calculation of the mean average precision (mAP) it includes the recall as well. Since the mean average precision is calculated based on an 11-point interpolated precision-recall curve, which is generated using different thresholds for confidence score, the confidence score is somewhat integrated in the metric. However, the model output evaluated still depends on what the user selects or uses as the default confidence score threshold. Combining this with the limited number of bounding boxes used in evaluation (max. is 100), the incentive to minimize the false positives is low when using this limit. Furthermore, multiple predicted bounding boxes per ground-truth bounding are not punished as false positives as long as their IoU score satisfied the IoU threshold used for evaluation. This is a change compared to the Pascal VOC mAP calculation in which these bounding boxes are penalized.

In this paper, we consider only the mAP which is averaged with IoU thresholds between 0.5 and 0.95 at steps of. 0.05. The procedure would be the same if e.g., the mean average precision of small objects (mAP_small_) would be of interest for selecting a model.

In contrast, the (PDQ Probability-based Detection Quality), introduced by Hall et al. [30], focuses on a probabilistic evaluation what an object detected is and where it is. The main reason for choosing this metric is that the PDQ explicitly penalizes false positives and false negatives and penalizes low confidences scores. Furthermore, it assesses and includes an average label quality and an average spatial quality and therefore the PDQ explicitly evaluates whether a model is better at locating or classifying an object.

Similar to the Pascal VOC mAP, the PDQ is calculated as the best match between the ground-truth labels and the detections. Bounding boxes that are not considered a best match are counted as false positives.

The label quality is assessed based on a categorical distribution over all class labels. The spatial quality is measured as a probability distribution of individual pixels against the ground-truth annotation mask and not the ground-truth bounding box. Therefore, predicted bounding boxes are not evaluated against ground-truth bounding using the standard IoU metric. Considering an object of non-rectangular shape parts of this object are likely to be occupy more space inside a bounding box than other parts. If a detection is centered more around this part, it will be scored higher than if the detection covers other parts of the bounding box with a smaller amount for foreground (=object) pixels.

For our purposes we use the PDQ as a proxy as the standard computer vision models for object detection do not output the required input for PDQ evaluation, namely quantifying spatial and label uncertainty of a bounding box. We converted the standard COCO-style predictions to a format suitable for computing the PDQ (https://github.com/david2611/pdq_evaluation, accessed on 26 February 2021, to convert coco results and compute the PDQ).

An in-depth analysis of more common object detection model performance evaluation metrics can be found in Padilla et al. [47].

### 2.4. Evaluation Process

All models are evaluated on their respective dataset without filtering the model output by a minimum confidence score if applicable. The output is evaluated using the standard COCO metrics and the PDQ with minimum confidence score thresholds ranging from 0.0 to 0.95 in steps of 0.05. If a model uses non-maximum suppression, the default IoU threshold of a model was used and not changed.

## 3. Results

### 3.1. COCO Dataset

**Figure 2:**
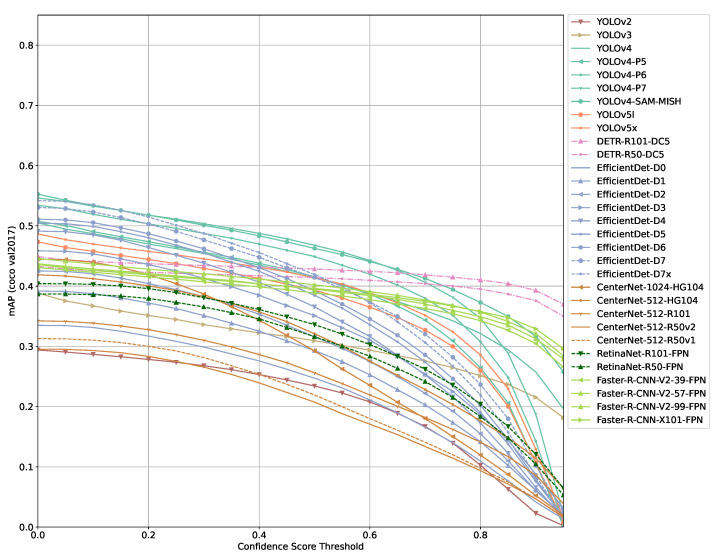
COCO mAP results of models selected (bigger models).

**Figure 3:**
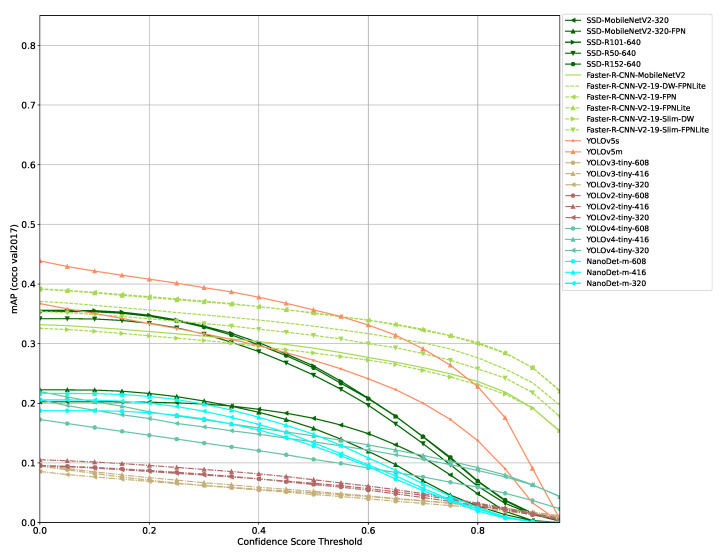
COCO mAP results of models selected (smaller models).

Evaluating the models selected using the COCO evaluation API for different confidence score thresholds leads to similar results for all models (Figure 2 and Figure 3). The mAP of a model decreases monotonically with increasing threshold values. The gradient of mAP drops varies from model to model and with confidence score threshold ranges. At some threshold value, a more significant drop is observable. Other COCO metrics (mAP_small_, mAP_medium_, mAP_large_, etc.) show similar results though individual models are ranked differently than in the mAP comparison.

Although most models mAP score drop to (close to) zero mAP at a confidence score threshold of 0.95, there are a few exceptions. Most notably DETR and models using Faster R-CNN. These models, especially DETR, show a more constant mAP curve with much lower gradients from low to high confidence score thresholds.

With lower confidence thresholds, a model outputs a lot more false positives than if a higher threshold is used. This indicates that the COCO evaluation score seems to not penalize false positives that much.

**Figure 4:**
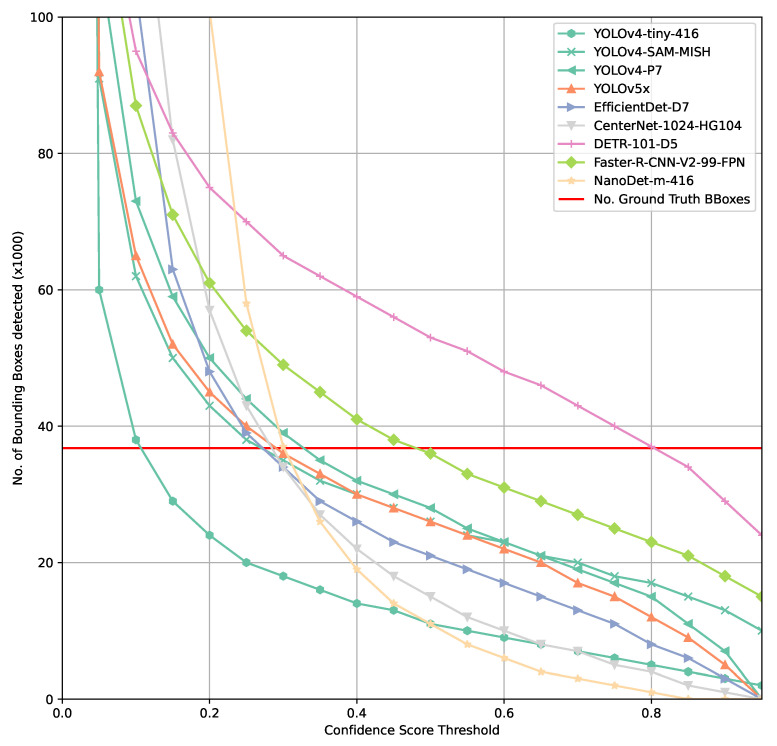
Comparison of number of bounding boxes predicted by selected models.

This becomes apparent when considering the total number of bounding boxes detected by a model on the COCO val2017 subset. Figure 4 already indicates fundamental challenges with finding an optimal confidence score threshold as well as evaluating a model’s performance. Most models show a rather steep gradient approaching the intersection between number of ground-truth bounding boxes across all classes in the COCO val2017 set and the number of predicted bounding boxes across all classes.

Assuming a perfect model, this intersection would be at a high confidence threshold and no false positives or false negatives would be found. In such a case, a very simplified approach to understand an optimal confidence score threshold would be based on the assumption that every lower confidence score threshold chosen would result in false positives whereas and higher threshold would result in false negatives.

Comparing Figure 2, Figure 3 and Figure 4 shows that there is a higher tolerance for false positives produced by a model than for false negatives. This clearly indicates that the model architectures and training processes involved are optimized for the COCO evaluation function as it penalizes false positives only marginally. In practice, a high and persistent false positives count can result in similarly poor performance than if a model is not able to detect objects of interest. A simple example for such a case would be an autonomous robot not able to perform its task since it might drop into to a safety state constantly because too many obstacles/objects that should not be harmed might be detected constantly. A similar adverse scenario might be some form of intrusion detection, either on premises or in orbit that either detects nothing or triggers so many false alarms that the human responsible to act on this alarm will simply ignore it.

**Figure 5:**
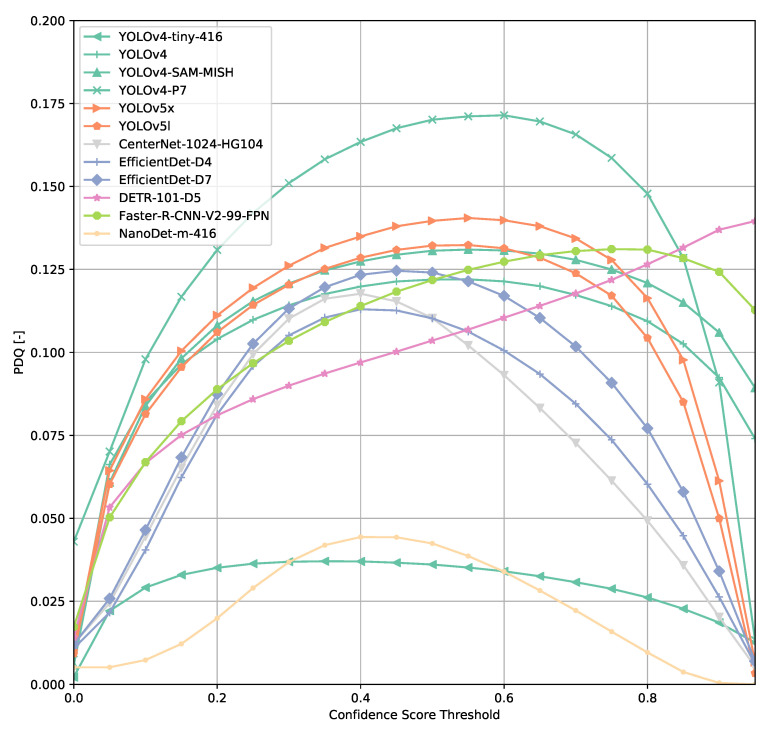
PDQ curves of selected subset of models.

Computing the PDQ as a function of confidence score threshold leads to a different kind of curve than the COCO metrics (Figure 5). Using such a PDQ curve, we are ending up with a confidence score threshold that could be understood as the optimal operating point of a model. Since the PDQ penalizes false positives and false negatives and incentivizes higher confidence scores attached to a prediction, the curve could be understood as follows. If a confidence score threshold lower than for PDQ_max_ is used, false positives are predominantly penalized, whereas choosing a higher threshold leads to significant penalization of a model’s performance due to an increasing number of false negatives.

**Figure 6:**
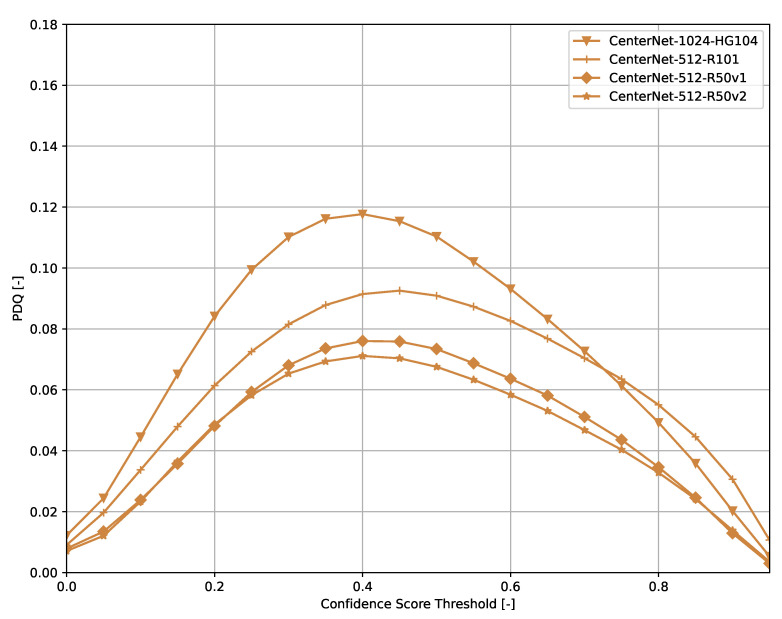
PDQ results of CenterNet on the COCO dataset.

**Figure 7:**
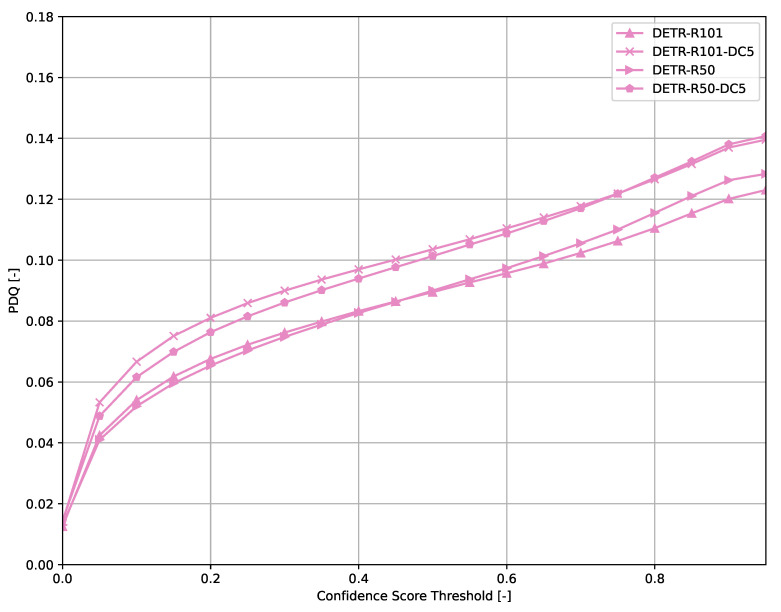
PDQ results of DETR on the COCO dataset.

**Figure 8:**
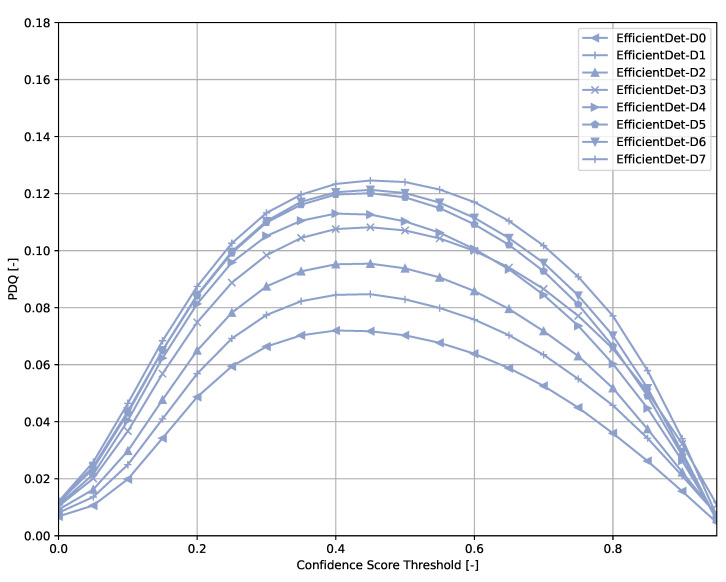
PDQ results of EfficientDet on the COCO dataset.

**Figure 9:**
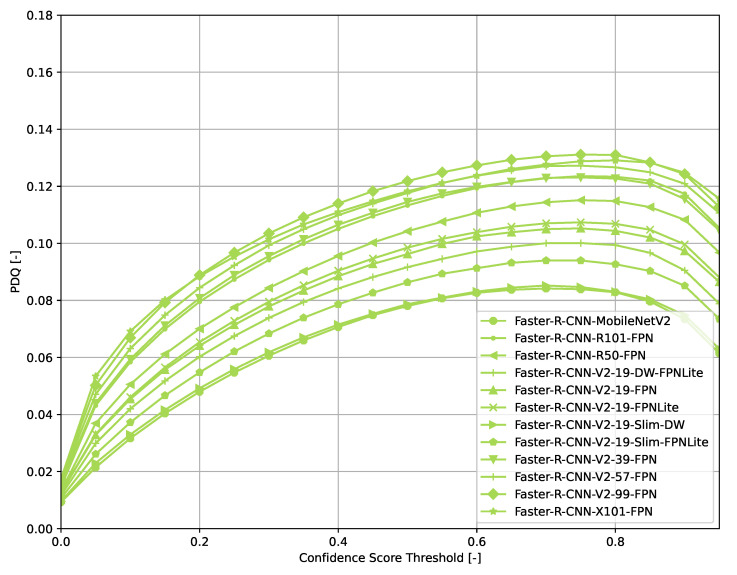
PDQ results of Faster R-CNN on the COCO dataset.

**Figure 10:**
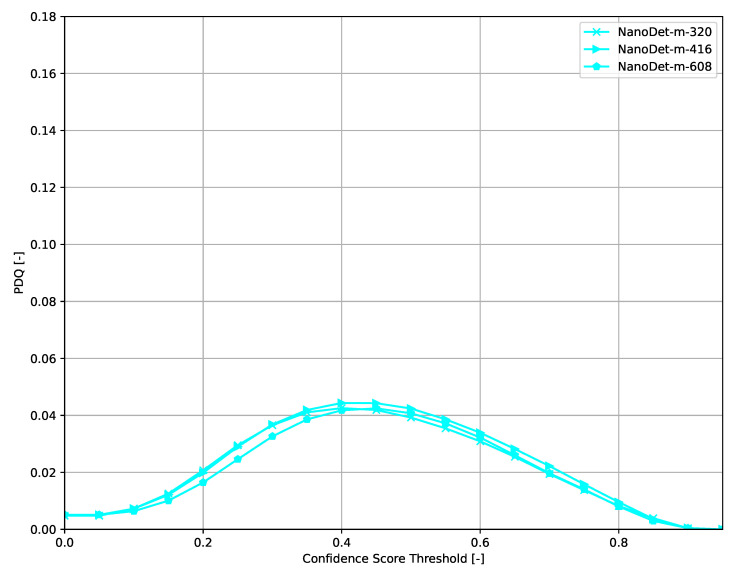
PDQ results of NanoDet on the COCO dataset.

**Figure 11:**
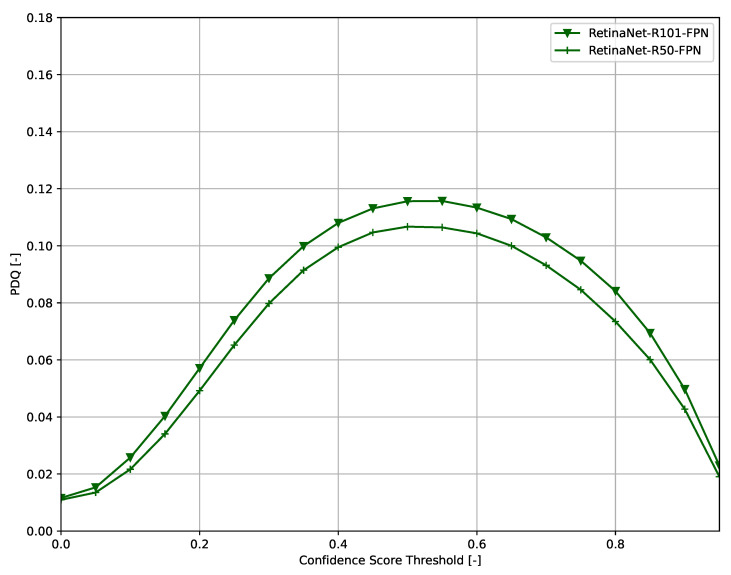
PDQ results of RetinaNet on the COCO dataset.

**Figure 12:**
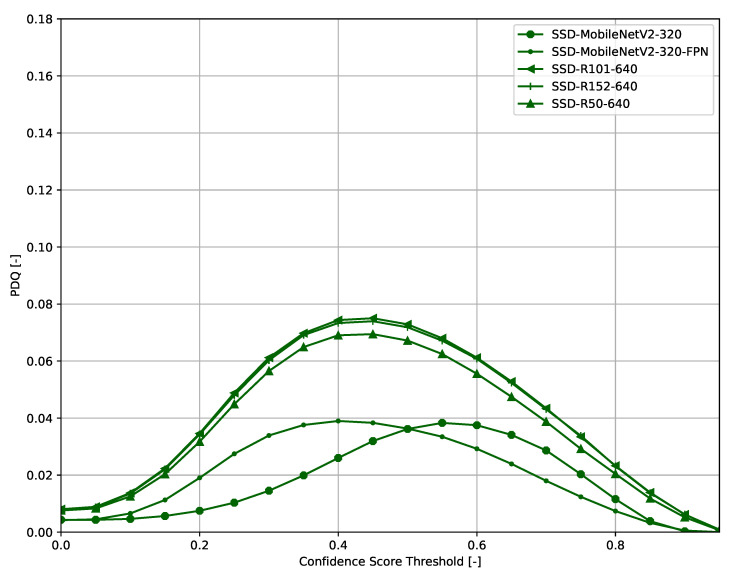
PDQ results of SSD on the COCO dataset.

**Figure 13:**
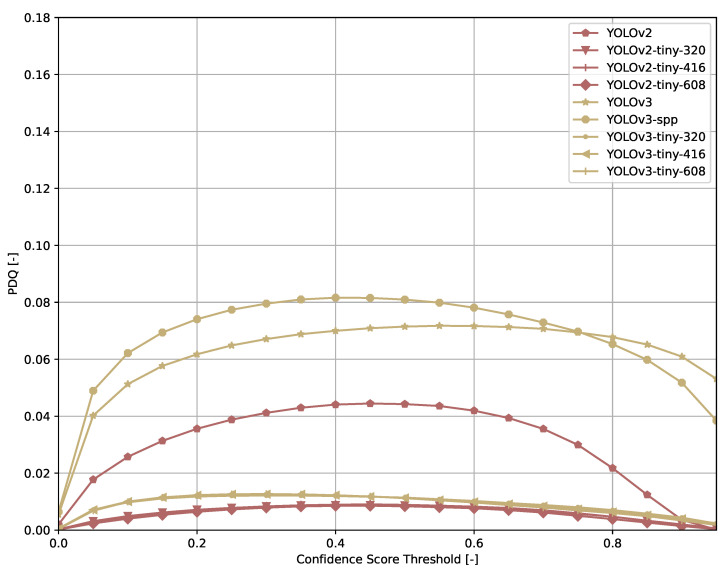
PDQ results of YOLOv{2,3} on the COCO dataset.

**Figure 14:**
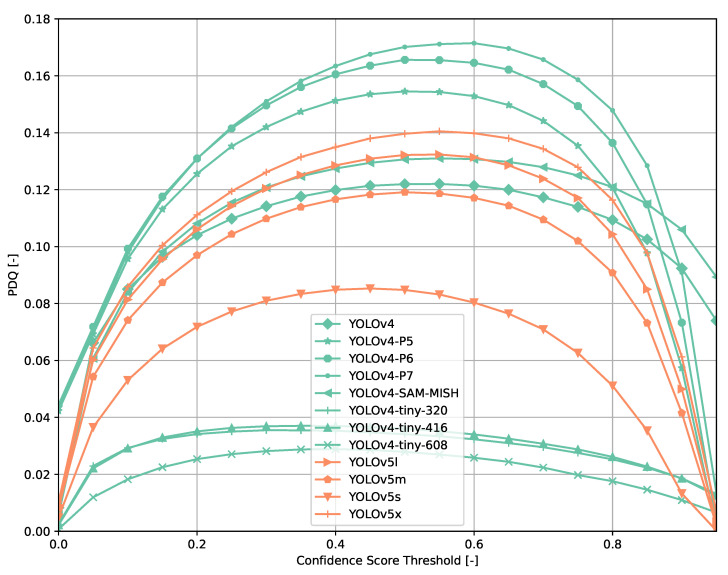
PDQ results of YOLOv{4, 5} on the COCO dataset.

Across all models (Figure 6, Figure 7, Figure 8, Figure 9, Figure 10, Figure 11, Figure 12, Figure 13 and Figure 14), PDQ curves seem to peak at a slightly higher confidence score threshold than at the respective number of bounding box intersection curves. This implies that the false negatives count is higher than the false positives count when selecting a model at PDQ_max_.

An exception to the general trend of (a)symmetric hill-shaped PDQ curves are the DETR models (Figure 7). These models output a large number of bounding boxes even at threshold of 0.8 and therefore, the trade-off between false positives and false negatives is still rather low when using a high confidence score threshold. Faster R-CNN-based models also show higher confidence thresholds than most other models evaluated.

The PDQ allows the gaining of more insights into a model’s performance than the COCO evaluation score. A significant observation is that most models have a high average label quality, often above 80%, whereas the average label quality is a lot lower (Table 2). The highest average spatial quality observed at PDQ_max_ is still below 25%. Therefore, we can conclude that the models evaluated are a lot more suited to tell what an object is and are less suited to tell us where exactly it is. We must point out that partially these low scores might also originate from the format conversion to evaluate COCO-style bounding boxes using the PDQ. However, no model evaluated in the original PDQ publication exceeds an average spatial score of 45% [30].

### 3.2. TTPLA Dataset

**Figure 15:**
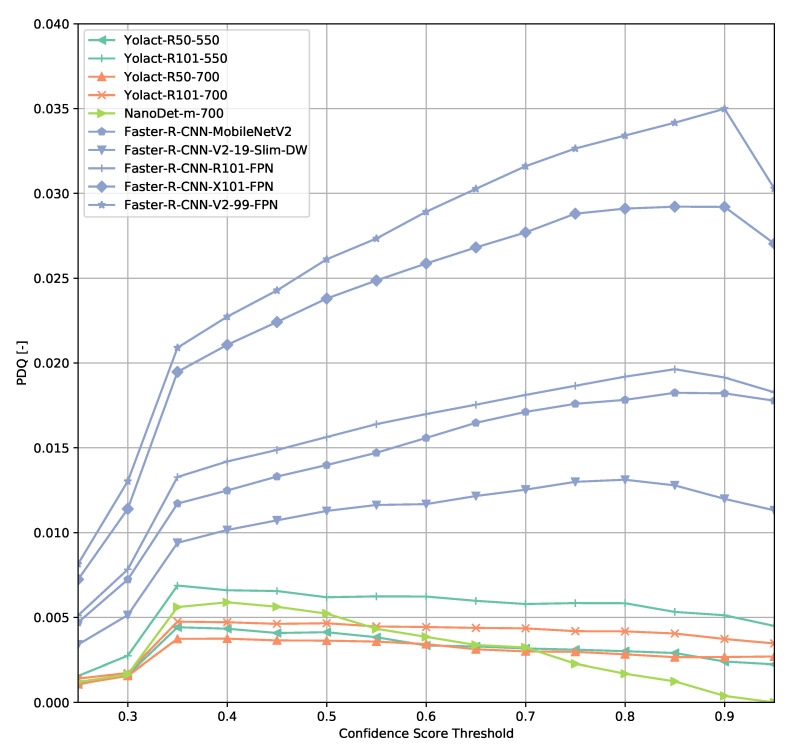
PDQ results on TTPLA.

**Figure 16:**
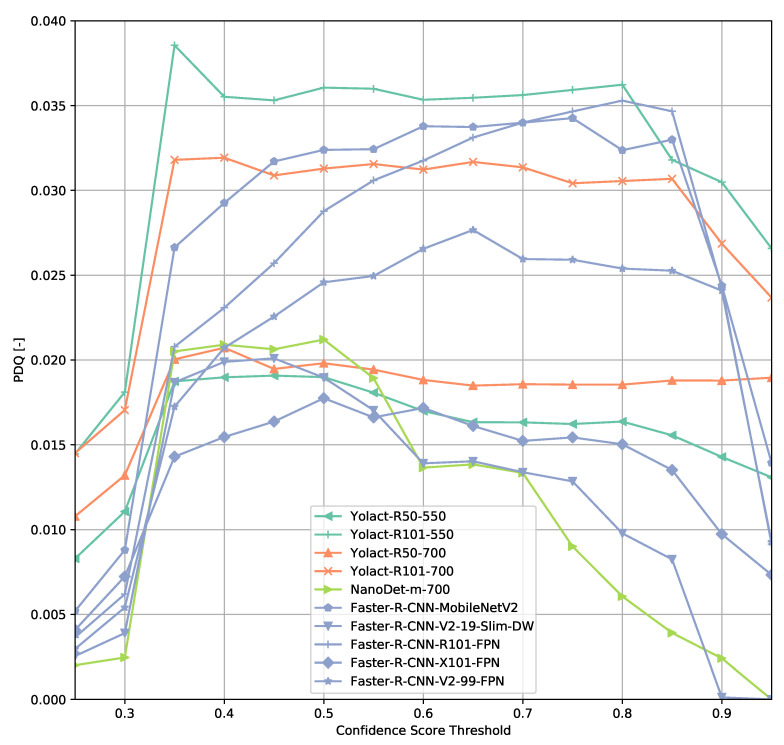
PDQ results on TTPLA (power lines excluded from evaluation).

The results on the TTPLA dataset are similar to the results on the COCO validation set (Figure 15 and Figure 16). Since TTPLA contains power lines which are objects of with a high aspect ratio, the object detection task is more challenging than on the COCO dataset. This is reflected in the PDQ scores which are a lot lower compared to the ones obtained on the COCO dataset. The shape of the PDQ curves seem to be similar when looking at the Faster R-CNN-based models whereas e.g., NanoDet shows a peak shifted to lower confidence score thresholds.

However, excluding the power line predictions from evaluation indicates that Faster R-CNN-based models seem to generalize a lot better on this dataset than e.g., the ResNet-based YOLACT reference models as the PDQ values change less though finding the optimal confidence score threshold seems to be trickier depending on the model architecture used. This could be caused by filtering out power line detections from evaluation and not retraining a model without power line objects as the transmission towers are easier objects to detect.

## 4. Discussion

The results indicate that there is at least one better way to evaluate a model’s performance than using the COCO evaluation metrics. Furthermore, we propose a PDQ-backed method to select a suitable confidence score threshold to find a model’s optimal performance.

However, such an optimal setting still comes with trade-offs. Except for the smaller models and a few larger models, we end up with a TP/FN ratio ranging from 0.8 to 1.4 for most models at PDQ_max_. Therefore, we must acknowledge that the number of correctly detected models and the total amount of missed objects is approximately of the same order of magnitude. Depending on the deployment case for a particular model, this might have severe consequences and implications when selecting a model for deployment.

In this paper, we did not compare model performance at PDQ_max_ against FPS explicitly. However, as a rule of thumb, smaller and faster models perform significantly worse than larger models. Depending on application-specific requirements when engineering a “tiny” model for edge device deployment, our findings should be taken into consideration.

In a more general setting, the method proposed solves only a subset of challenges involved with training and comparing models for object detection. False positives and missed detections might highly fluctuate based on small changes between consecutive frames. This might be primarily caused by the issue that most neural network architectures overfit to texture [48] and are susceptible to small pixel shifts [49]. To some extend this texture bias can be used for adversarial attacks as well [50,51,52].

In this paper, we focus on model performance on the COCO and TTPLA datasets. On different datasets, model performance may vary lot as most of the models evaluated were developed and designed with the COCO metric as the main design goal. The ranking of models implied by Figure 5 may change depending on what another dataset a model is trained on and how it is trained on a certain dataset. Since we used pre-trained models provided by reference implementations, we cannot estimate the impact of training method variations on the results presented in this paper.

Other data driven approaches to estimate model performance or verify a model’s performance, e.g., a test scenario based on real data or simulated might profit from a more rigorous metric and model selection process as well.

It might be of further interest to investigate the effects of using different datasets with significantly less classes but larger variability of objects per class as we would expect for e.g., an autonomous robot. As most models published are designed for a few benchmark datasets, it is of interest how the confidence score threshold for PDQ_max_ may change on other datasets. Furthermore, it would be of interest to investigate how to achieve a better average spatial score and how a potential integration of the method propose into a model’s design and training process would lead to more robust models.

## 5. Related Work

To the best of our knowledge, this is the first paper evaluating the impact of selecting confidence score thresholds rigorously using standard models for object detection. In a less rigorous way, this approach was used to find the optimal performance of a single model [53] for a PDQ-related computer vision competition.

Using f1-scores (e.g., [54]) or similar metrics as a function of confidence score is not helpful either as these approaches depend on selecting IoU thresholds for evaluation as well. Any f1 score will depend on selecting IoU thresholds and selecting confidence scores.

Furthermore, using f1 scores with the FP, FN, and TP counts as provided by the PDQ will lead to confidence score thresholds shifted towards lower thresholds as it only takes FPs, FNs, and TPs into consideration. Higher confident scores of predictions are not incentivized.

## 6. Conclusions

In this paper, we introduce a simple approach to find the optimal confidence score threshold for fairer model performance comparison. We evaluated a subset of state-of-the-art models using this approach. At this proposed operating point the trade-offs between counts of true positives, false positives negatives, and the overall confidence in bounding boxes detected are minimized. Furthermore, we would like to point out that many models show a high average label quality but a poor average spatial quality. Therefore, there is a lot of room for developing better models that can use latest advancements in sensor technology to provide useful products and services to customers. With respect to edge devices (e.g., UAV-based, static cameras or orbital sensors), these deployment scenarios are only feasible if models deployed output predictions with sufficiently good spatial and label qualities. Deploying models with non-optimal settings renders such applications pointless as the trade-offs between false negatives, false positives and true positives are too high.

## Figures and Tables

**Table 1:** Models selected for evaluation.

Model	Reference	Implementation _(accessed on 15 January 2021)_
CenterNet	[35]	https://github.com/tensorflow/models
DETR	[36]	https://github.com/facebookresearch/detr
EfficientDet	[37]	https://github.com/google/automl/
Faster R-CNN-MobileNetV2	-	https://github.com/youngwanLEE/vovnet-detectron2
Faster R-CNN-Resnet	-	https://github.com/facebookresearch/detectron2
Faster R-CNN-ResNeXt	-	https://github.com/facebookresearch/detectron2
Faster R-CNN-VOVNETv2	[38,39]	https://github.com/youngwanLEE/vovnet-detectron2,
NanoDet	-	https://github.com/RangiLyu/nanodet,
RetinaNet	[40]	https://github.com/tensorflow/models,
RetinaNet-FPN	-	https://github.com/facebookresearch/detectron2
SSD (MobileNetv2-320)	-	https://github.com/tensorflow/models
YOLOv2(-,-tiny)	[41]	https://github.com/AlexeyAB/darknet
YOLOv3(-,-tiny,-SPP)	[42]	https://github.com/AlexeyAB/darknet
YOLOv4(-,-SAM-MISH)	[31]	https://github.com/AlexeyAB/darknet
YOLOv4-tiny	[43]	https://github.com/AlexeyAB/darknet
YOLOv4-P(5-7)	[43]	https://github.com/WongKinYi/ScaledYOLOv4
YOLOv5(s,m,l,x)	[44]	https://github.com/ultralytics/yolov5

**Table 2:** COCO metrics comparison at PDQ_max_.

Model	Confidence	PDQ_(max)_	Spatial	Label	mAP_(max)_	mAP	∆mAP	mAP50	mAP75	mAPs	mAPm	mAPl	TP	FP	FN
	Threshold		(PDQ)	(PDQ)											
	[%]	[%]	[%]	[%]	[%]	[%]	[p.p.]	[%]	[%]	[%]	[%]	[%]			
CenterNet-1024-HG104	40	11.77	21.18	64.03	44.47	34.33	10.14	44.61	38.29	19.80	36.08	44.60	17,590.	4611	19,191
CenterNet-512-R101	45	9.26	17.75	69.31	34.23	27.28	6.96	37.81	29.74	8.10	29.58	44.49	14,781	4169	22,000
CenterNet-512-R50v1	40	7.60	14.72	62.74	31.29	25.35	5.94	36.62	27.87	8.67	27.46	40.44	15,257	6563	21,524
CenterNet-512-R50v2	40	7.11	14.64	62.84	29.53	23.94	5.60	34.39	26.17	7.10	26.07	39.46	13,840	5287	22,941
DETR-R101	95	12.30	17.07	97.41	43.49	35.62	7.87	49.74	38.69	15.14	39.11	53.25	18,136	5194	18,645
DETR-R101-DC5	95	13.96	19.54	97.58	44.90	37.01	7.89	50.81	40.13	16.52	40.74	54.52	18,791	5374	17,990
DETR-R50	95	12.83	18.86	97.44	42.01	33.97	8.04	47.63	36.65	14.18	36.76	52.36	17,387	4553	19,394
DETR-R50-DC5	95	14.07	20.50	97.34	43.27	35.01	8.27	48.59	37.92	15.61	38.65	51.38	18,179	5062	18,602
EfficientDet-D0	40	7.20	13.38	66.97	33.48	27.58	5.91	39.02	30.90	6.00	31.04	47.84	14,249	4567	22,532
EfficientDet-D1	45	8.47	15.03	70.46	39.18	30.92	8.26	41.75	34.64	8.08	35.40	50.42	14,720	3538	22,061
EfficientDet-D2	45	9.54	15.92	70.31	42.52	33.51	9.01	44.65	37.20	12.71	37.71	52.97	16,034	3586	20,747
EfficientDet-D3	45	10.82	16.60	71.75	45.87	36.75	9.12	48.14	40.75	16.15	40.65	55.66	17,634	4065	19,147
EfficientDet-D4	40	11.30	16.65	68.44	49.13	40.64	8.49	53.35	45.07	20.22	45.33	58.99	19,239	4897	17,542
EfficientDet-D5	45	12.01	17.56	71.36	50.45	40.47	9.99	52.52	44.76	21.42	44.67	57.60	18,921	4178	17,860
EfficientDet-D6	45	12.13	17.05	71.67	51.10	41.26	9.85	53.27	45.74	21.98	45.12	59.22	19,631	4415	17,150
EfficientDet-D7	45	12.46	17.37	72.67	53.07	43.05	10.01	55.37	47.32	23.77	47.07	60.67	19,762	4521	17,019
Faster-R-CNN-MobileNetV2	70	8.42	12.63	89.85	33.18	25.98	7.20	39.08	28.90	11.97	27.15	36.25	16,379	6658	20,402
Faster-R-CNN-R101-FPN	75	12.36	16.67	92.84	42.42	34.86	7.55	49.06	39.04	17.06	38.59	48.15	19,174	6048	17,607
Faster-R-CNN-R50-FPN	75	11.51	15.81	92.61	40.52	33.09	7.42	47.39	37.27	15.32	36.52	46.14	18,523	6168	18,258
Faster-R-CNN-V2-19-DW-FPNLite	75	10.01	14.14	91.95	37.08	29.10	7.98	42.89	32.73	14.71	31.54	39.32	17,233	5423	19,548
Faster-R-CNN-V2-19-FPN	75	10.53	14.30	92.20	39.20	31.32	7.88	45.60	34.98	16.34	33.77	42.13	18,209	6011	18,572
Faster-R-CNN-V2-19-FPNLite	75	10.74	14.76	92.41	39.13	31.34	7.79	45.69	34.98	16.39	33.99	42.66	18,132	5900	18,649
Faster-R-CNN-V2-19-Slim-DW	70	8.52	12.50	89.81	32.57	25.52	7.05	39.01	28.46	11.73	26.88	35.16	16,518	6380	20,263
Faster-R-CNN-V2-19-Slim-FPNLite	75	9.40	13.67	91.61	35.40	27.22	8.18	40.51	30.36	13.71	28.41	37.40	16,732	5594	20,049
Faster-R-CNN-V2-39-FPN	75	12.31	16.29	93.06	43.09	35.41	7.69	50.12	39.65	18.61	38.85	47.75	19,408	5916	17,373
Faster-R-CNN-V2-57-FPN	75	12.72	16.64	93.16	43.70	35.97	7.73	50.74	40.10	18.97	39.61	49.25	19,689	6010	17,092
Faster-R-CNN-V2-99-FPN	75	13.11	17.02	93.45	44.59	36.78	7.81	51.50	41.09	18.85	39.97	50.98	19,808	5781	16,973
Faster-R-CNN-X101-FPN	80	12.91	17.20	94.55	43.58	35.78	7.80	49.87	40.13	18.26	38.89	49.07	19,171	5485	17,610
NanoDet-m-320	40	4.25	11.75	57.35	20.57	16.45	4.12	25.70	17.47	2.21	14.40	30.02	10,322	5150	26,459
NanoDet-m-416	40	4.44	10.76	57.34	21.65	17.66	3.99	28.12	18.70	4.08	17.47	28.52	11,974	7035	24,807
NanoDet-m-608	45	4.25	10.60	59.03	18.75	14.29	4.47	23.72	14.79	5.32	17.00	19.42	11,542	6058	25,239
RetinaNet-R101-FPN	55	11.57	19.38	78.03	40.41	32.03	8.38	44.15	35.67	13.92	35.88	44.42	16,700	4287	20,081
RetinaNet-R50-FPN	50	10.67	17.83	75.03	38.69	31.61	7.08	44.32	35.20	14.11	34.80	44.27	17,285	5899	19,496
SSD-MobileNetV2-320	55	3.83	9.79	66.54	20.24	16.35	3.89	25.98	17.43	1.13	11.26	36.49	10,357	7059	26,424
SSD-MobileNetV2-320-FPN	40	3.90	9.55	58.11	22.25	18.49	3.76	29.07	20.22	1.39	17.91	35.03	10,793	5683	25,988
SSD-R101-640	45	7.50	14.61	64.01	35.60	28.28	7.32	39.60	31.83	8.28	30.93	45.02	14,440	4553	22,341
SSD-R152-640	45	7.40	14.39	64.11	35.40	27.99	7.42	39.10	31.36	8.16	30.22	45.63	14,302	4587	22,479
SSD-R50-640	45	6.94	13.82	63.35	34.19	26.79	7.40	37.92	30.25	8.22	28.63	43.29	13,961	4613	22,820
YOLOv2	45	4.45	6.63	68.93	29.39	24.41	4.98	42.13	25.69	6.44	28.16	40.53	14,358	5053	22,423
YOLOv2-tiny-320	45	0.88	2.81	67.46	9.54	6.96	2.57	15.69	5.16	0.33	4.64	15.40	5957	8631	30,824
YOLOv2-tiny-416	45	0.90	2.47	66.90	10.53	7.71	2.82	17.75	5.52	0.67	6.75	15.55	6774	9287	30,007
YOLOv2-tiny-608	45	0.86	2.20	64.86	9.59	6.84	2.74	16.93	4.18	1.66	9.16	10.04	7263	9127	29,518
YOLOv3	55	7.18	9.01	88.61	38.84	30.16	8.68	48.99	33.48	16.69	33.11	42.04	17,811	6670	18,970
YOLOv3-spp	40	8.16	10.68	78.20	42.59	33.20	9.39	49.47	38.02	16.96	34.60	48.09	18,121	5426	18,660
YOLOv3-tiny-320	30	1.21	3.85	62.36	8.56	6.23	2.33	11.27	6.46	0.02	1.98	19.19	6513	6668	30,268
YOLOv3-tiny-416	30	1.26	3.57	60.18	9.65	6.68	2.97	12.35	6.49	0.04	4.75	18.52	7423	7047	29,358
YOLOv3-tiny-608	30	1.27	3.59	58.37	9.46	6.20	3.26	11.95	5.67	0.24	9.48	11.93	7811	7261	28,970
YOLOv4	55	12.20	15.72	86.27	50.50	40.13	10.38	54.63	46.24	23.10	46.01	53.27	19,103	3896	17,678
YOLOv4-P5	50	15.45	22.74	77.57	50.75	41.55	9.20	53.43	46.13	21.68	47.06	56.69	20,163	4926	16,618
YOLOv4-P6	50	16.56	23.34	78.62	53.41	44.91	8.49	57.42	49.51	25.83	50.12	60.84	21,455	5451	15,326
YOLOv4-P7	60	17.15	24.58	82.58	54.63	44.25	10.38	55.55	48.65	22.50	50.34	62.37	20,053	3862	16,728
YOLOv4-SAM-MISH	55	13.10	15.42	88.02	55.26	45.23	10.02	61.09	52.34	29.02	51.41	59.89	20673	4097	16,108
YOLOv4-tiny-320	30	3.55	7.45	68.66	20.55	16.03	4.52	27.78	16.73	2.70	17.48	28.83	10,706	5263	26,075
YOLOv4-tiny-416	35	3.70	7.10	70.73	21.97	16.53	5.44	28.64	17.23	4.80	19.91	24.84	11,366	4961	25,415
YOLOv4-tiny-608	40	2.89	6.38	67.50	17.28	12.06	5.22	22.28	11.76	5.86	17.89	11.92	10,486	6737	26,295
YOLOv5l	55	13.24	20.22	78.53	47.34	38.04	9.29	49.62	42.26	18.11	44.25	52.79	18,470	4283	18,311
YOLOv5m	50	11.91	18.51	76.45	43.88	35.66	8.22	47.87	39.45	17.07	41.59	49.02	18,166	4882	18,615
YOLOv5s	45	8.53	14.67	71.02	36.71	28.49	8.23	40.43	31.97	12.44	33.75	38.64	15,788	4614	20,993
YOLOv5x	55	14.05	20.71	79.32	48.66	40.35	8.31	52.55	44.51	20.59	46.16	56.40	19,463	4770	17,318

## Data Availability

Not applicable.
